# Supplementary material for: Perceptions and Experiences of the University of Nottingham Pilot SARS-CoV-2 Asymptomatic Testing Service: A Mixed-Methods Study
Source: Int J Environ Res Public Health. 2020 Dec 29;18(1):188. doi: 10.3390/ijerph18010188 (PMC7796111; doi:10.3390/ijerph18010188)
Supplement: Supplementary file 1 [file ijerph-18-00188-s001.zip › Supplementary files/Supplementary File 1 COREQ_Consolidated criteria for reporting qualitative studies .docx]

**Supplementary Table 2. Consolidated criteria for reporting qualitative studies (COREQ): 32-item checklist**

| **Topic and Item No.** | **Guide Questions/Description** | **Response** |
| --- | --- | --- |
| **Domain 1: Research team and reflexivity** | | |
| *Personal Characteristics* | | |
| 1. Interviewer/facilitator | Which author/s conducted the interview or focus group? | Holly Blake and Cecilia Cirrelli |
| 2. Credentials | What were the researcher’s credentials? E.g. PhD, MD | Holly Blake: PhD  Cecilia Cirelli: Medical trainee |
| 3. Occupation | What was their occupation at the time of the study? | HB: health psychologist/university academic;  CC: medical trainee/ university research assistant |
| 4. Gender | Was the researcher male or female? | 2 female |
| 5. Experience and training | What experience or training did the researcher have? | HB: mixed methods researcher, experienced interviewer and  focus group moderator, GCP trained;  CC: qualitative research training during research assistant positions. GCP trained |
| *Relationship with participants* | | |
| 6. Relationship established | Was a relationship established prior to study commencement? | Researchers met the participants during recruitment |
| 7. Participant knowledge of the  interviewer | What did the participants know about the researcher? e.g. personal goals, reasons for doing the research | Participants knew that HB was a university academic, and that CC was a medical trainee. They knew that both interviewers were affiliated to the participating university but that they were not from the same School as the participants. |
| 8. Interviewer characteristics | What characteristics were reported about the interviewer/facilitator? e.g. Bias, assumptions, reasons and interests in the research topic | Participants knew that HB and CC were interested in the experiences of students during the COVID-19 pandemic. |
| **Domain 2: Study design** |  |  |
| *Theoretical framework* | | |
| 9. Methodological orientation and  Theory | What methodological orientation was stated to underpin the study? e.g. grounded theory, discourse analysis, ethnography, phenomenology, content analysis | Thematic analysis |
| Participant selection | | |
| 10. Sampling | How were participants selected? e.g. purposive, convenience, consecutive, snowball | Purposive (e.g. gender, living circumstances (on or off campus), first or final year of study). |
| 11. Method of approach | How were participants approached? e.g. face-to-face, telephone, mail, email | Participants were approached and recruited by email |
| 12. Sample size | How many participants were in the study? | 41 participants took part in qualitative interviews or focus groups (with free text qualitative responses from a sample of 99 survey respondents) |
| 13. Non-participation | How many people refused to participate or dropped out? Reasons? | 11 signed up to interviews or focus groups but did not attend due to other commitments. No participants actively withdrew. |
| *Setting* | | |
| 14. Setting of data collection | Where was the data collected? e.g. home, clinic, workplace | Data were collected online (video-conferencing platform) |
| 15. Presence of non-participants | Was anyone else present besides the participants and researchers? | Interviews: No.  Focus Groups: An administrator was present at 3 of the group interviews to act as scribe. |
| 16. Description of sample | What are the important characteristics of the sample? e.g. demographic data, date | Students: Year of study, gender.  Staff: Job role. |
| *Data collection* | | |
| 17. Interview guide | Were questions, prompts, guides provided by the authors? Was it pilot tested? | Questioning guide was shared with members of a university operations and management board for the ETTP.  In developing the guide, input was sought from virologists, students and university academic staff. The guide was pilot tested with students from a different cohort. |
| 18. Repeat interviews | Were repeat interviews carried out? If yes, how many? | No repeat interviews. |
| 19. Audio/visual recording | Did the research use audio or visual recording to collect the data? | Interviews and focus groups were audio-recorded using a video-conferencing platform. Interviewers kept cameras on to assist with establishing rapport. |
| 20. Field notes | Were field notes made during and/or after the interview or focus group? | Yes. |
| 21. Duration | What was the duration of the interviews or focus group? | Interviews: approx. 60 mins  Focus groups: approx. 60-90 mins. |
| 22. Data saturation | Was data saturation discussed? | Yes. |
| 23. Transcripts returned | Were transcripts returned to participants for comment and/or correction? | Yes - a sub-sample. |
| **Domain 3: analysis and findings** |  |  |
| *Data analysis* | | |
| 24. Number of data coders | How many data coders coded the data? | Two. |
| 25. Description of the coding tree | Did authors provide a description of the coding tree? | No, however initial coding was informed by the interview guide, and coding was continuously  refined. |
| 26. Derivation of themes | Were themes identified in advance or derived from the data? | These were derived from the data. |
| 27. Software | What software, if applicable, was used to manage the data? | N’Vivo |
| 28. Participant checking | Did participants provide feedback on the findings? | Yes. |
| *Reporting* | | |
| 29. Quotations presented | Were participant quotations presented to  illustrate the themes / findings? Was each  quotation identified? e.g. participant number | Yes. |
| 30. Data and findings consistent | Was there consistency between the data  presented and the findings? | Yes. |
| 31. Clarity of major themes | Were major themes clearly presented in the  findings? | Yes. |
| 32. Clarity of minor themes | Is there a description of diverse cases or discussion of minor themes? | Yes. |
