# Supplementary material for: Perceptions and Experiences of the University of Nottingham Pilot SARS-CoV-2 Asymptomatic Testing Service: A Mixed-Methods Study
Source: Int J Environ Res Public Health. 2020 Dec 29;18(1):188. doi: 10.3390/ijerph18010188 (PMC7796111; doi:10.3390/ijerph18010188)
Supplement: Supplementary file 1 [file ijerph-18-00188-s001.zip › Supplementary files/Supplementary File 4 Thematic map illustrating the relationships between the key themes and subthemes.docx]

**Supplementary File 6:** Thematic Map showing relationships between the key themes and subthemes
