# Supplementary material for: Perceptions and Experiences of the University of Nottingham Pilot SARS-CoV-2 Asymptomatic Testing Service: A Mixed-Methods Study
Source: Int J Environ Res Public Health. 2020 Dec 29;18(1):188. doi: 10.3390/ijerph18010188 (PMC7796111; doi:10.3390/ijerph18010188)
Supplement: Supplementary file 1 [file ijerph-18-00188-s001.zip › Supplementary files/Supplementary File 2 Post-Programme Student Evaluation Survey.pdf]

Student Survey SVMS 76-0720

Page 1

You were recently invited to take part in an Enhanced Testing Pilot for COVID-19. We would like to ask you some questions to better understand your view of university community testing for COVID-19, in order to inform future approaches to COVID-19 outbreak prevention and management.

The survey is open to any student that was *invited* to take part in the SVMS COVID-19 Enhanced Testing Pilot whether you chose to take part, or not.

Page 2

ABOUT YOU

In this first section, we are going to ask several questions about you. NONE of these answers will be shared with any third parties.

1. How old are you? (please enter a whole number) ✱ *Required*

2. What was your gender at birth? ✱ *Required*

- ○ Male
- ○ Female
- ○ Other
- ○ Prefer not to say

3. What is your ethnicity? ✱ *Required*

- ○ White
- ○ Mixed
- ○ Asian or Asian British
- ○ Black or Black British
- ○ Middle Eastern or Middle Eastern British
- ○ Other Ethnic groups
- ○ Prefer not to say

3.a. Are you...

- British
- Irish
- Any other white background

3.b. Are you...

- White and black Caribbean
- White and black African
- White and Asian
- Any other mixed background

3.c. Are you...

- Indian
- Pakistani
- Bangladeshi
- Any other Asian background

3.d. Are you...

- Caribbean
- African
- Any other Black background

3.e. Are you...

- Chinese
- Any other ethnic group

3.f. Are you...

- Arab
- Turkish
- Other

4. Are you an international student? (i.e., not from within the UK?) ✱ *Required*

- Yes, I'm from the EU
- Yes, I'm from outside EU/UK
- No, I'm from the UK

4.a. Please indicate which country you are from:

5. What stage of study are you at? ✱ *Required*

- Foundation course/Certificate
- Registered for single modules only
- 1st year of undergraduate study
- 2nd year of undergraduate study
- 3rd year of undergraduate study
- 4th year of undergraduate study
- 5th year or higher of undergraduate study
- I am a postgraduate

6. Do you have any caring responsibilities (e.g., child/ adult family member)? ✱ *Required*

- ◯ Yes
- ◯ No
- ◯ Prefer not to say

7. Do you have a history of anxiety, depression or any other mental health issue for which you have received treatment in the past? ✱ *Required*

- ◯ Yes
- ◯ No
- ◯ Prefer not to say

8. Do you have an existing physical health issue that you think could affect your risk of getting COVID-19? ✱ *Required*

- ◯ Yes
- ◯ No
- ◯ Prefer not to say

9. Where are you living at the moment during term time? ✱ *Required*

- ◯ Private accommodation on own
- ◯ Private accommodation with family
- ◯ Private accommodation (with others)
- ◯ Hall of residence

9.a. Which hall of residence are you living in?

- Lenton and Wortley Hall
- Cripps Hall
- Hugh Stewart Hall
- Derby Hall
- Nightingale Hall
- Rutland Hall
- Ancaster Hall
- Sherwood Hall
- Beeston Hall
- Cavendish Hall
- Florence Boot Hall
- Willoughby Hall
- Lincoln Hall
- Newark Hall
- Southwell Hall
- Broadgate Park (excluding Albion/Cloister House)
- Albion House
- Cloister House
- Dagfa House
- Nottingham Two
- Raleigh Park
- Riverside Point
- St Peter’s Court
- Melton Hall
- Varsity
- Bonington Student Village
- Derby University Student Living Halls
- Partnership Halls in Lincoln

9.b. How many members are there in your current household, including yourself? (by household, we mean the people you live with)

- 1
- 2
- 3
- 4
- 5
- 6
- 7 or more

9.b.i. How many of them are children under 16 years old?

10. How do you travel to campus usually? (Please select the most frequent method you use) ✱ *Required*

- On foot
- Cycling
- Bus (city transport)
- University Hopper Bus
- Tram
- Train
- Car
- Other

10.a. If you selected Other, please specify:

11. Are you doing paid or voluntary work during term time? ✱ *Required*

- ◯ Yes
- ◯ No

11.a. Does this work bring you into close contact with others? By 'close contact' we mean people who are within 2 metres of you.

- ◯ No
- ◯ Yes, but I always or nearly always wear a face covering
- ◯ Yes, but I only wear a face covering some of the time

11.b. On a typical day, my work brings me into close contact (i.e., within 2 metres) with...

- ◯ <5 people
- ◯ 5-10 people
- ◯ >10 people

11.c. If you work/volunteer, how do you usually travel to get there? (Please select the most frequent method you use)

- ◯ On foot
- ◯ Cycling
- ◯ Bus (City transport)
- ◯ University Hopper Bus
- ◯ Tram
- ◯ Carpool
- ◯ Other
- ◯ N/A – No travel required, remote work

11.c.i. If you selected Other, please specify:

12. Are you currently fulfilling any of the government's identified 'key worker' roles? *
Required

- Health, social care or relevant related support worker
- Teacher or childcare worker still travelling in to work
- Transport worker still travelling in to work
- Food chain worker (e.g. production, sale, delivery)
- Key public services worker (e.g. justice staff, religious staff, public service journalist or mortuary worker)
- Local or national government worker delivering essential public services
- Utility worker (e.g. energy, sewerage, postal service)
- Public safety or national security worker
- Worker involved in medicines or protective equipment production or distribution
- Other 'key worker' role not listed
- None of these

Page 3

ABOUT COVID-19

In this section, we are going to ask you some questions relating to COVID-19.

13. Have you had COVID-19 symptoms previously? (By symptoms we mean one or more of the following: a fever, new continuous cough, or loss of taste or smell) ✱ *Required*

- Yes
- No

14. Have you ever had a COVID-19 test? (the test which tells whether you have COVID-19 currently) ✱ *Required*

- Yes, once
- Yes, more than once
- No

14.a. Thinking about your most recent test, when were you tested?

- December 2019
- January 2020
- February 2020
- March 2020
- April 2020
- May 2020
- June 2020
- July 2020

- August 2020
- September 2020
- October 2020

14.b. Thinking about your most recent test, what was the result of your test?

- Positive for COVID-19
- Negative for COVID-19
- Not sure
- Prefer not to say

14.c. My COVID-19 test was undertaken...

- At the university
- Elsewhere
- Both

14.d. Which country or countries had you visited in the 6 months prior to being tested for COVID-19?

15. Have you been **shielding** at any time during the pandemic? (By '**shielding**' we mean being **advised to** protect yourself from COVID-19 because you or a member of your household/bubble are **considered high risk**. Staying at home as much as possible and taking extra precautions and avoiding contact with people outside the household (but still able to go outside for essential travel and daily exercise) ✱ *Required*

- Yes
- No

15.a. For what reason(s) have you been shielding?

- My own health reason(s)
- To protect the health of others
- Both of the above
- Prefer not to say

16. Have you had to **self-isolate** previously? By '**self-isolate**' we mean staying at home and avoiding contact with people outside the household in order to protect others as you may be at risk of passing on COVID-19 ✱ *Required*

- Yes, once
- Yes, more than once
- No

16.a. When did you have to **self-isolate**? (If you isolated more than once, state the most recent time)

- December 2019
- January 2020
- February 2020
- March 2020
- April 2020
- May 2020
- June 2020
- July 2020
- August 2020
- September 2020
- October 2020

16.b. Why did you have to **self-isolate**? (select all that apply)

- ☐ I experienced COVID-19 symptoms but I didn't have a test.
- ☐ I tested positive for COVID-19.
- ☐ Someone in my household/bubble tested positive for COVID-19.
- ☐ Someone in my household/bubble had symptoms and did not have a test.
- ☐ I came into contact with someone outside of my household/bubble who tested positive for COVID-19.
- ☐ Other (e.g., return from travel to high risk area)

16.c. Which statement best describes your experience of self-isolation (select one)

- ○ I stayed in my own room and had no contact with anyone else.
- ○ I mostly stayed in my own room but had some contact with other people in my household/bubble (e.g. eating meals together).
- ○ I stayed in my own house but did not stay in my room (e.g. had regular contact with people in my household/bubble but did not have contact with anyone outside the house).
- ○ I did not self-isolate (e.g. I had contact with others outside my household/bubble).

16.d. If you had to **self-isolate**, were you satisfied with the University support during this time?

- ○ Extremely satisfied
- ○ Somewhat satisfied
- ○ Somewhat dissatisfied
- ○ Extremely dissatisfied

16.d.i. Tell us more:

17. Do you know anyone that (select all that apply): ✱ *Required*

- ☐ Had (or think they have had) COVID-19
- ☐ Had to be in hospital with COVID-19
- ☐ Died from COVID-19
- ☐ None of the above

18. On a scale of 1-10, BEFORE THE PILOT TESTING PROGRAMME, what did you believe your risk of getting COVID-19 was? ✱ *Required*

- ○ 1 (I didn’t think I would get it)
- ○ 2
- ○ 3
- ○ 4
- ○ 5
- ○ 6
- ○ 7
- ○ 8
- ○ 9
- ○ 10 (I knew I will most certainly get it)

19. On a scale of 1-10, what do you currently believe your risk of getting COVID-19 is? ✱ *Required*

- ○ 1 (I don't think I will get it)
- ○ 2
- ○ 3

- 4
- 5
- 6
- 7
- 8
- 9
- 10 (I know I will most certainly get it)

20. Please read the following statements carefully and then select the one which best describes how you have felt over the past 2 weeks. ✱ *Required*

- I do not worry about getting COVID-19
- I occasionally worry about getting COVID-19
- I spend much of my time worrying about getting COVID-19
- I spend most of my time worrying about COVID-19

21. Please read the following statements carefully and then select the one which best describes how you have felt over the past 2 weeks. ✱ *Required*

- I do not worry about my close relative(s)/friend(s) getting COVID-19
- I occasionally worry about my close relative(s)/friend(s) getting COVID-19
- I spend much of my time worrying about my close relative(s)/friend(s) getting COVID-19
- I spend most of my time worrying about my close relative(s)/friend(s) getting COVID-19

22. On a scale of 1-10, how important do you think social distancing is in controlling the spread of the virus? By social distancing we mean trying to stay at least 2 metres (3

steps) away from anyone who is not in your household/bubble. ✱ *Required*

- 1 (not at all important)
- 2
- 3
- 4
- 5
- 6
- 7
- 8
- 9
- 10 (very important)

23. On a scale of 1-10, how important do you think regular hand washing is in controlling the spread of the virus? ✱ *Required*

- 1 (not at all important)
- 2
- 3
- 4
- 5
- 6
- 7
- 8
- 9
- 10 (very important)

24. On a scale of 1-10, how important do you think self-isolating is in controlling the spread of the virus? ✱ *Required*

- 1 (not at all important)
- 2
- 3
- 4
- 5
- 6
- 7
- 8
- 9
- 10 (very important)

25. On a scale of 1-10, how important do you think wearing a face covering is in controlling the spread of the virus? ✱ *Required*

- 1 (not at all important)
- 2
- 3
- 4
- 5
- 6
- 7
- 8
- 9
- 10 (very important)

26. If you were offered a COVID-19 vaccine, would you take it? ✱ *Required*

- Yes

- No
- Unsure

27. Did you take part in the 'SVMS COVID-19 pilot testing programme? ✱ *Required*

- Yes
- No

27.a. Please share your reasons:

27.b. In this section, we will ask you about your experiences of the SVMS pilot testing programme. Did you access support from your personal tutor with relation to the pilot testing programme?

- Yes
- No

27.c. How many swabs did you complete as part of this pilot?

- None
- 1
- 2
- 3
- 4
- 5
- 6
- 7

- 8
- 9
- 10
- 11
- 12

27.c.i. If you didn't complete all of the swab tests you were asked to complete, why not?

27.d. How many saliva tests did you complete as part of this pilot programme?

- None
- 1
- 2
- 3
- 4
- 5

27.d.i. If you didn't complete all of the saliva tests you were asked to complete, why not?

27.e. How did you usually collect your swab or saliva kits?

- collected myself
- collected by others in household/bubble

- mix of both
- N/A - didn't complete any tests

27.f. Were you able to return the test kit by the requested time?

- Yes, always
- Yes, sometimes
- No, never
- N/A - didn't complete any tests

27.g. Were you satisfied with the location of the drop-off?

- Yes
- No
- N/A - didn't drop off any kits myself

27.h. Were you satisfied with the university approach to communicating positive test results? By this, we mean a text and phone call for anyone testing positive.

- Yes
- No

27.h.i. Tell us why:

27.i. Were you generally satisfied with the way in which negative swab or saliva test results were communicated to you?

- Yes

- No

27.i.i. Tell us why:

27.j. Did you receive a positive swab or saliva test result from the University testing service during this pilot?

- Yes
- No

27.j.i. On average, how long after you dropped off your swab or saliva test kit were you contacted by the Clinical Virologist (text and/or phone call) with your positive result?

- Same day
- Next day
- Two days or more

27.j.ii. Did you notify the University of your positive result using the online reporting form?

- Yes – same day
- Yes – next day or later
- No

27.j.iii. How long after notifying the University using the online form did you receive the official test kit which those testing positive in the pilot are asked to complete?

- Same day

- Next day or later
- Didn’t receive an official test kit

27.k. How many antibody tests (finger-prick) did you complete as part of this pilot?

- None
- 1
- 2
- 3
- 4
- 5
- 6 or more

27.k.i. If you didn’t complete all the antibody tests, why not?

27.l. How did you collect your antibody test kits (finger-prick)?

- collected myself
- collected by others in household/bubble
- mix of both
- didn’t collect an antibody test kit

27.m. Were you able to return the completed antibody test kit by the requested time?

- Yes, always
- Yes sometimes
- No, never
- N/A - didn't complete any antibody tests

27.n. Were you satisfied with the location of the drop-off?

- Yes
- No
- N/A - didn’t drop off a completed kit myself

27.o. On average, how long after you dropped off your first completed finger-prick test kit did you receive the antibody test result?

- Same day
- Next day
- Two days later
- 3 or more days later
- N/A - didn’t receive a result
- N/A - didn't complete any antibody tests

27.o.i. Tell us more:

27.p. **Your overall views of the programme** In general, I felt satisfied with the information received.

- very satisfied
- somewhat satisfied
- somewhat dissatisfied
- very dissatisfied

27.q. In general, I felt satisfied with the way in which information was communicated to me.

- very satisfied
- somewhat satisfied
- somewhat dissatisfied
- very dissatisfied

27.r. I have gained new knowledge as a result of taking part in this pilot.

- Yes
- No

27.r.i. If yes, what have you learned?

27.s. Self-testing using a swab was acceptable to me.

- Yes
- No

27.s.i. If no, why not?

27.t. Self-testing using a saliva test was acceptable to me.

- Yes
- No

27.t.i. If no, why not?

27.u. Antibody testing using a finger prick was acceptable to me.

- Yes
- No

27.u.i. If no, why not?

27.v. I was confident in the outcome of my test results.

- Yes
- No

27.v.i. If no, why not?

Page 4

Your overall views of the programme (continued)

28. The pilot testing programme has had the following benefits (select any that you agree with)

- ☐ Being able to contribute to national efforts to control COVID-19
- ☐ Helping to keep campus safe for everyone
- ☐ Learning something new about COVID-19
- ☐ Learning something new about COVID-19 testing procedures
- ☐ Getting to know other students better
- ☐ Getting to know university staff better
- ☐ Being involved in COVID-19 research
- ☐ Having pride in my School
- ☐ Having pride in the University of Nottingham

28.a. Are there any other benefits?

29. Is there anything about the pilot testing programme that has been difficult or less positive?

30. I feel safer to be present at the university knowing people are being tested for COVID-19 ✱ *Required*

- Strongly agree
- Somewhat agree
- Somewhat disagree
- Strongly disagree

30.a. Tell us why?

31. Our local communities will be safer because people at the university are being tested for COVID-19 ✱ *Required*

- Strongly agree
- Somewhat agree
- Somewhat disagree
- Strongly disagree

31.a. Tell us why?

32. I feel reassured about my own health status knowing people are being tested for COVID-19 ✱ *Required*

- Strongly agree
- Somewhat agree
- Somewhat disagree
- Strongly disagree

32.a. Tell us why?

33. My family will be reassured knowing people at the university are being tested for COVID-19 ✱ *Required*

- Strongly agree
- Somewhat agree
- Somewhat disagree
- Strongly disagree

33.a. Tell us why?

34. I would take part in university testing for COVID-19 in the future if it was offered to me. ✱ *Required*

- Yes

- No
- Unsure

35. I would encourage others to take part in university testing for COVID-19 if it was offered to them. ✱ *Required*

- Yes
- No
- Unsure

Your well-being

Over the last two weeks, how often have you been bothered by any of the following problems?

36. Feeling nervous, anxious or on edge? ✱ *Required*

- Not at all
- Several days
- More than half the days
- Nearly every day

37. Not being able to stop or control worrying? ✱ *Required*

- Not at all
- Several days
- More than half the days
- Nearly every day

38. Worrying too much about different things? ✱ *Required*

- Not at all
- Several days
- More than half the days
- Nearly every day

39. Trouble relaxing? ✱ *Required*

- Not at all
- Several days
- More than half the days
- Nearly every day

40. Being so restless that it is hard to sit still? ✱ *Required*

- Not at all
- Several days
- More than half the days
- Nearly every day

41. Becoming easily annoyed or irritable? ✱ *Required*

- Not at all

- Several days
- More than half the days
- Nearly every day

42. Feeling afraid as if something awful might happen? ✱ *Required*

- Not at all
- Several days
- More than half the days
- Nearly every day

43. Is there anything else you would like to tell us?

44. If you would be willing to take part in a focus group or short interview by Microsoft Teams or telephone to share your experience in more detail, please provide your consent here. **I consent to take part in an interview or focus group**: ✱ *Required*

- Yes
- No

44.a. What is the email address that you would like us to use to contact you to arrange this? This will NOT be shared with any third parties. Contact details will be separated from your questionnaire replies prior to analysis. Valid university email address:

Please enter a valid email address.

@nottingham.ac.uk

Page 5: Final page

This is the end of the survey.

Thank you for your time, your views are very important to us.
