# Supplementary material for: Perceptions and Experiences of the University of Nottingham Pilot SARS-CoV-2 Asymptomatic Testing Service: A Mixed-Methods Study
Source: Int J Environ Res Public Health. 2020 Dec 29;18(1):188. doi: 10.3390/ijerph18010188 (PMC7796111; doi:10.3390/ijerph18010188)
Supplement: Supplementary file 1 [file ijerph-18-00188-s001.zip › Supplementary files/Supplementary File 5 Key themes, subthemes and quotes.docx]

**Supplementary File 7.** Themes, subthemes and their representative quotes

| **Theme** | **Subthemes** | **Representative quotations** |
| --- | --- | --- |
| **Emotional Responses to COVID-19** | Negative Feelings | I am definitely suffering from COVID fatigue, that is how much I think about COVID (P126, Female, Staff – clinical academic)  In the beginning when it all went into lockdown it felt really alien and it made me quite anxious (P128, Female, Staff – academic)  I think it has been a lot of like adaptation but I think the most stressful part was when I came back and had to go on rotations because then you couldn’t avoid the thing that was stressing you, which was like seeing other people. (P101, Female, Yr5, Student).  I kind of thought it was a bit inevitable like I knew obviously everyone is moving from all over around the country and like because we were here in the summer and then we saw all of the September freshers move in. They are all bringing like three people each, it was kind of inevitable. It didn’t surprise me but at the same time like it didn’t really increase my concern either because I just kind of thought like I can only protect myself, so it doesn’t affect me that much. (P111, Female, Yr1, Student)  In the first few weeks I wasn’t like scared of it but I was unsure of it, just because I didn’t know how I felt about it and I couldn’t work it out… I couldn’t live with myself if I had given it to anybody (P116, Female, Yr1, Student)  I kind of thought it was going to get worse than people had imagined so I didn’t go to Uni the two weeks before that as well. Because I was a bit like oh I don’t know if people have it, I don’t know how serious it is, so I am just going to like stay away from people (P101, Female, Yr5, Student) |
|  | COVID-19 Around Me | My eldest son has Asthma and I mean he is nine, so he is young, but it was still a concern that I was thinking crikey if he gets COVID is that going to be a massive deal? (P128, Female, Staff – academic)  A lot of people that I know, recently in particular who have tested positive for COVID, the symptoms have been very mild, like normally just a scratchy throat, just a bit of a cough and the more people that you see that are presenting signs like that, the less seriously people are to take the erm the whole pandemic in general. (P106, Female, Yr5, Student)  Erm, my parents are both over 60, my mother in particular was shielding, I opted to stay at university, both for exams and for lockdown purposes (P106, Female, Yr5, Student)  I had not left my house since March and so I didn’t know anyone who had had COVID or been out anywhere that I could get COVID really, so I was quite nervous moving here. (P111, Female, Yr1, Student)  We’re taking it quite seriously because we have people in our bubble who were shielding because they have got underlying health conditions like asthma, erm but erm they are not shielding anymore they are just taking the risks by going on rotations and people haven’t been taking it very seriously on rotations (P119, Female, Yr5, Student)  One of my friends was on a ventilator for ten weeks because of the erm being affected by COVID, he was a nurse working with elderly people so that was quite a scary experience (P126, Female, Staff –academic) |
|  | Coping with COVID-19 | Sometimes if like there is more cases on the news and stuff it makes you feel a little bit worried about it but I think it is better being at uni with my friends and stuff erm yes it has definitely made me feel better being like surrounded with all of my friends (P91, Female, Yr1, Student)  Sometimes I feel so much better if I just don’t watch the news and not even think about it because you turn the news on for a bit in the morning and it is just a bit depressing (P91, Female, Yr1, Student)  Yes, I think isolation was all right, it was just erm trying to keep busy really. Not get too bored (P105, Male, Yr1, Student)  I think probably since post lockdown it is not that I am more relaxed about it, I still take all of the necessary precautions but I am far less anxious about it and I do wonder if it is because out of sort of a bit out of my control. (P127, Female, Staff – academic)  “We have had situations where students have significant mental health crises during self-isolation and that has put a lot of stress on the other students in the house where they are in an isolating situation… we need to make sure we have enough support 24 hours a day, seven days a week, for these students” (P129, Male, Staff – academic). |

Theme 2

| **University Life during COVID-19** | Adapting to a new normal | I don’t keep two metres, I am going to be brutally honest with you, when it is with close friends because not only do I trust them to tell me if they have caught it, there is other things I have got balance it, I am going to go insane if I don’t see people (P116, Female, Yr1, Student)  It is just something that I kind of am living with and taking a different course of action in my life to deal with but it doesn’t cause me that much anxiety anymore (P128, Female, Staff – academic)  I was reading about like how people’s views towards coronavirus had changed, and like, because they had so much fear in the beginning, they have kind of adjusted to that level and then they will still go out and they will still socialise, even though if they thought about doing that like a month ago, that would have terrified them. I think everybody has adjusted to like the new normal (P101, Female, Yr5, Student)  I think we have kind of accepted the fact that we’re likely to get it now because most people like the numbers are increasing with the positive cases and also it is likely that we’re going to go in to Tier three (P112, Female, Yr1, Student)  I did my first semester completely online from home. It is possible… And actually, I think I got more done at home than I do here. (P116, Female, Yr1, Student).  You kind of get used to studying online and then you go there, and you get used to studying there, and then you come back and then you’re thinking like you are virtually (P117, Female, Yr1, Student).  I think there is a fear as well, there is a fear of not making friends at uni. So, when people come here, everyone wants to make lots of friends because that is where you’re here to do at uni, so it is kind of hard having those restrictions kind of prevent that. (P98, Female, Yr1, Student)  I think we probably shouldn’t have come back to uni if they were expecting us to be just on our own. That is not going to happen, because otherwise you’re going to have a lot of mental health problems, so I think that is unnecessary (P94, Female, Yr1, Student)  If you’re in a household where you don’t really get along or you don’t really socialise and you have the option to go and see I don’t know the football team or some friends that you went to university with or somebody is having a get together and you don’t want to feel left out. It is much more challenging to say no in those types of situations. (P106, Female, Yr5, Student)  Yes, I felt anxious at first coming back to work, but I was really keen to get back to work (P123, Female, Staff – Senior administrator)  Self isolating yes is difficult, but it is definitely…. it is different as well for different people with gardens like you still need to be able to get sunlight, sunlight is really important erm if you can’t open the window because it has got a safety lock on and you’re on the ground floor (P117, Female, Yr1, Student)  Because a lot of my release is going outside, riding horses and just going for walks and stuff and when you can’t do that, and you don’t really even have a window to lean out of. It drives you a bit nuts. (P107, Female, Yr1, Student)  There was no way we could have come out of this erm in a good light because either we would have been the generation that was not you know supporting the economy when it was needed or we went out and people got sick (P106, Female, Yr5, Student)  I think in a way when it was just us April students, I think the spread of COVID on the campus was lower so maybe it is the fact that more students have come back that it is a higher spread. Which is kind of what is said in the media but then again I think also like people going to pubs that are not students, I think they are just as to blame as us. I don’t feel like we’re the sole reason for it spreading, I feel like there is other people as well. (P112, Female, Yr1, Student) |
| --- | --- | --- |
|  | Improving University Life | Means of having food delivered is a big one, potentially access to some sort of entertainment, be it you know a subscription package or… or like just some form of entertainment. And I think they are the big ones, people get bored and people feel that they need to go out and get stuff and if you have that then… the people that will adhere would adhere much more happily. (P106, Female, Yr5, Student)  It doesn’t need to be a lot, simple email, not even every day like every other day, how are you doing? Do you need anything? Things like that… I am sure providing some sort of like cleaning products or hand gel, even not like loads just hand sanitiser or something that you could put in the flat, to help protect them would be quite nice. (P116, Female, Yr1, Student)  Just helping to make the university a safer place and just keeping erm COVID levels as low as possible by making people isolate… I think all we need is like shopping deliveries and any post etcetera. I think that is all in place as it is (P112, Female, Yr1, Student)  Just send me food like if just at least once a day, genuinely like some of the girls I was with had real problems, real problems and I have different eating requirements (P117, Female, Yr1, Student)  I know they [University] are taking people’s bins out for them and taking deliveries in for them and bringing them to their door kind of thing, so I suppose definitely doing stuff like that. Erm, just yes just making sure that people don’t have to leave their flat for anything and if they can take food deliveries in and just make sure everyone has got what they need really. (P121, Female, Yr1, Student)  Just making it convenient so it doesn’t clash with lectures and make it like out of hours or since doing them myself anyway, yes maybe you could offer an incentive if you really wanted to go there like erm around a prize draw every month or something but I don’t think you need to do that but that would encourage me anyway (increasing swabbing uptake) (P119, Female, Yr5, Student)  “So, some support around, for the students, around what happens when you get a positive result would be extremely useful to come alongside that [the testing], so that did increase our workload quite considerably.” (P129, Male, Staff – academic). |

Theme 3

| **Influences on Testing Participation** | Testing Freedom | I just wanted to know if I was positive so that I could take steps not to spread it and to kind of know that I was you know at risk and people near me were at risk so that was my main driver (P128, Female, Staff – academic)  I think it just put my mind at rest really when we were going home to see family and if we were going back to Nottingham we didn’t want to be like spreading it everywhere. It made it easier for us to like live a bit more normally. (P94, Female, Yr1, Student)  Similar reasons to be honest and it just means you’re comfortable going to practicals without sort of being so worried all of the time. (P98, Female, Yr1, Student)  I have opted just to do the testing for my own peace of mind (P106, Female, Yr5, Student) |
| --- | --- | --- |
|  | External Influences | I guess you could say I was influenced by the university really pressing us to do it as a good idea (P100, Male, Yr1, Student)  We were encouraged quite a lot by the vet school, I know we received emails about it and they were like oh yes if you would like to take part in this, they were like very… they encouraged us quite a lot to do it (P107, Female, Yr1, Student)  I did it because all of my flat did it and we just decided that we would do it together, erm and also I just wanted to help out and be part of the research (P112, Female, Yr1, Student)  Everybody in my flat was doing it and I was like OK I need to do it because everybody else is doing it and I am going to look odd if I am not doing it (P116, Female, Yr1, Student)  Yes, like an activity so you would feel left out if you didn’t do it (P93, Female, Yr1, Student)  Mine was mainly a personal choice just to make sure but also some of our rotations require us to have a test before they will let us on the premises (P109, Female, Yr5, Student) |
|  | Curiosity | We are scientists really and I think we should be doing these things, we should be pushing ourselves, we should be seeing what we can do and how we can do it so curiosity but also you know we were the very first people who actually designed something like that and went through the pain because there was loads of pain on behalf of people who are doing it. It is not you know it wasn’t that easy to take off, so I think that is something to be proud of. (P126, Female, Staff – Clinical academic)  I thought the study was quite interesting so I guess the interest would drive me to continue doing the study. (P94, Female, Yr1, Student)  Oh, I loved finding out how it worked because I came back, and I am able to talk about it more. I am able to sort of say you know this isn’t as deadly to us as we think and quite for better or worse we are white so it doesn’t seem to have a more deadly affect that it is having (P117, Female, Yr1, Student)  I just wanted to know whether I like had it or whether I have got it whilst I would be here especially because we’re the first people to go back to uni so it is quite like interesting to find out how the whole situation is going to run (P91, Female, Yr1, Student) |

Theme 4

| **Testing Physical and Logistical Factors** | Communication | Yes, it was good, we didn’t get told when we had negative results but then we got an email at the end of the day saying that anyone who was positive had been contacted so obviously you knew that you were negative. Erm which I think worked absolutely fine like I don’t think you need to be notified if you’re negative if there is a lot of people doing the study, I guess it takes a lot of time. (P121, Female, Yr1, Student)  It has been quite consistent throughout at the beginning it was very clear about what we needed to, when we needed to do it, erm and yes I think it has been… it was all done by email which is good because it means everyone can access it. (P92, Female, Yr1, Student)  I guess nothing was hidden from us like all of the information was given to us and from what I have seen when people are positive they literally get like a call within… as soon as they get tested positive. So, like and if you’re not positive they put it on an email to everyone that has not been positive this week or whatever, so it is quite clearly given to us. (P99, Male, Yr1, Student)  One of my main motivations was to get the antibody results, a) to make sure that I was free and to get the antibody results and that is where the communication has broken down because I still haven’t had the antibody results. And so, I was very tempted not to do anymore blood sticks because if we’re not going to get the results, why do it. (P129, Male, Staff – academic)  Yes, I think at one point they were only letting people who were positive know and you kind of assumed that if you didn’t hear, you were negative. But it was actually really nice getting that email to say that you were negative because otherwise there was always that slight question in your mind, did I remember to fill in the consent form? Or did my sample get lost or missed or something? So, it was actually really nice having that confirmation as an individual that you were negative. (P125, Female, Staff – academic)  I think it would have been better if you got an email every time, just because then you’re kind of like well did they do my test? Or did I just… like you’re just unsure there is always a little thing in the back of your head like did it actually test negative? Or did my test not get there? (P101, Female, Yr5, Student)  I was intrigued by the antibody testing that was available because that is still going on with the saliva samples but I did do the antibody testing and didn’t hear anything back (P106, Female, Yr5, Student)  I don’t know about antibody’s but with regards to that I… we still haven’t heard. Antibody testing, I did the one in the second week, I didn’t do the first one and I didn’t do the last antibody but I did every other one and I haven’t got a clue if I had or haven’t had COVID at any point. I think they are lost in the system somewhere. (P116, Female, Yr1, Student)  Would say when I first got the email it was very long erm and it was a bit confusing if I am honest, I didn’t really… I knew I got like the gist of erm like the testing erm when and sort of thing, but it was quite confusing but then as soon as I started like got the first test, it was really, really easy (P102, Male, Yr5, Student) |
| --- | --- | --- |
|  | Physical testing | I thought it all worked very well and I know some people were worried about sticking swabs in various different places, I didn’t think there was any problem with that at all (P129, Male, Staff – academic).  I personally felt that actually erm doing different tests was very useful for students, because they will understand how the animals feel when they are having certain things done so that was definitely something which I even considered that they should be doing (P126, Female, Staff – Clinical academic)  I found it fine, as I have said before I am diabetic, so I have my own finger pricker for the issues with that, the swab was fine, I never had an issue with it… preferred the swab erm for the simple fact that it was quicker. It is a surprising volume of saliva that you need (P106, Female, Yr5, Student)  He just said I don’t want to do it because first of all I know you stick it down your throat but then you put it up your nose (P117, Female, Yr1, Student)  I had a COVID test before I came here, and it was a lot easier doing myself erm and what we were asked to do I found it a lot easier doing it myself rather than someone else doing it. It makes you feel a bit more comfortable as well especially if your friends are doing it with you (P92, Female, Yr1, Student)  You can’t really get around the whole finger pricking thing because I know some people just don’t like the whole needle, getting stabbed aspect of that but the spitting in to a tube is kind of disgusting but I think it is easier than like the whole swab in the back of your throat (P91, Female, Yr1, Student)  The, erm, swab was a bit grim, it was yes quite difficult… well it wasn’t difficult it was just I wouldn’t say it was a pleasant experience. The saliva one was so much easier and so much better but yes I mean it was easy enough (P102, Male, Yr5, Student)  Yes I also found it really easy, erm I actually must just bleed a lot because I found the sticks absolutely fine and couldn’t work out what everyone else was on about erm until I had cold hands one day and then I really struggled to bleed myself (P127, Female, Staff – academic) |
|  | Practicalities of testing | I thought it was really easy, erm and erm we noticed because the drop off and the collection station is opposite our office, we noticed that the participation from the students, particularly the April cohort who are living on campus was really, really good. (P123, Female, Staff – Senior administrator)  The location was convenient because it was sort of on our way to most of our practical sessions so if we happened to have a practical that day we could drop them off on the way. (P94, Female, Yr1, Student)  I think it was pretty much all positive, the only thing was the timings. It was a bit annoying rushing through like some lecture or like running over to put them in before the deadline. (P94, Female, Yr1, Student)  I think it was a good amount of time, I also liked because at the time they were like… well at first they were like you need to come on this day or this day but I think by the end of it they were just like as long as you hand it in, we’re not really bothered. I liked that flexibility. (P116, Female, Yr1, Student)  I think once a week was OK to be fair. (P121, Female, Yr1, Student)  I think the weekly thing was just about right. (P100, Male, Yr1, Student)  I had one the swabs or the saliva and then they were just sat on the side and then nobody had time to go and get them so there were days where you would miss one (P130, Female, Staff – academic)  I missed the last two unfortunately so I haven’t done the saliva test and that was because there weren’t any erm on the days I was in and trying to do them (P128, Female, Staff – academic)  “I think it is very, very easy for us just to say oh yes you know somebody didn’t pick up the swabs or we run out of the swabs but the logistics were enormous… I think if we also had more communication about what is happening behind the scenes as you know the staff so we can understand better how much they are doing, I think maybe the process would just run a little bit more smoothly as well.” (P126, Female, Staff – Clinical academic) |

Theme 5

| **Testing Effects on Wellbeing** | Improved Mental Wellbeing | I think it has helped a lot with the kind of anxiety around, not really about myself getting COVID but I am always scared that if I was asymptomatic I would be giving it to other people so I wasn’t really scared for myself a lot of the time, I was scared that I would give it to other people and they would get sick and I just didn’t want that (P101, Female, Yr5, Student)  It was a good kind of confidence boost, you knew that it wasn’t going to spread around university as much as if people were asymptomatic and weren’t being tested. You knew that there was a bit more of a like safety net in a way (P121, Female, Yr1, Student)  I think it is just for kind of peace of mind it helped a bit, not having to worry about it all of the time and everyone said oh you’re going to university are you not worried? It was just kind of nice to know that there is awareness, and it is not just you know social distancing, you are actively trying to help as well I think. (P92, Female, Yr1, Student)  I think I just found it hugely reassuring, I was really, really keen to take part when I heard about that it was going to happen and I just… it made me feel well yes just that word it was just really reassuring to know that I was getting regularly tested and in a way I know you can’t directly say that everybody in my immediate family is OK but it was almost like I could act like the canary going down the mine and that there was a certain amount of reassurance as well that if I was negative there was a high chance that my children and my husband were also negative (P124, Female, Staff – Clinical academic) |
| --- | --- | --- |
|  | Behavioural change | I think we had two cases on campus in that time so when actually it was really safe and I think that really helped because when we did go out and you mixed, well I am probably going to be fine and I think everybody was a bit more chill then because they knew they were negative (P116, Female, Yr1, Student)  Yes I think erm there has been a few times when I have gone home erm just for the day to see my family and what not which I probably wouldn’t have done if the testing wasn’t in place so in that regard it has like changed my behaviour in sort of that way (P103, Female, Yr5, Student)  Yes I think it is really important, especially because the cases were already going up before people moved here and now there is a possibility of a second lockdown, it would make it a lot less likely if people were being tested and isolating before they spread it to potentially 100’s of people on campus. Because it could get to quite a scary situation if we just let it go… like ran ruckus around campus’. (P98, Female, Yr1, Student)  I mean I guess testing negative maybe made me a bit more comfortable to go to like go and play football and things like that but I am not a massive sort of go out person anyway. I was only really sort of the gym and that so… I guess it is a more sort of like oh it is all right I can go play football with a group of people and stuff but again I think [unclear 18:10] change too much because it was like the prospect of a potential positive test the week after so I think overall I probably stayed about the same really. (P105, Male, Yr1, Student)  Definitely reassuring erm that we’re getting tested and what not regularly erm it is also nice like if you do want to pop home just to see family for the day type thing, you know that you’re not going to be taking anything with you. I think if I didn’t have the testing I definitely wouldn’t be doing that (P103, Female, Yr5, Student)  I think for me personally it didn’t because I was still very conscious that like the test could be false negative and things like that but if I knew I was positive I wouldn’t go near anybody, but I felt confident going to see like my best friends but still at a distance and it didn’t like make me anxious… I don’t think it made me… I didn’t see it as like oh I got a negative test, now I can go and see everybody, definitely didn’t see it like that no. It just made me feel OK about seeing the people that I felt I needed to see to keep myself kind of happy and socially. But no, I definitely know some people who used it as like an excuse to party because they were negative so they could like behave terribly and go to parties and stuff which yes that would never cross my mind no. I think definitely some people but not me personally no. (P101, Female, Yr5, Student)  I would say generally in my house, most of the people, five out of six of us would be following the same rules anyway to be honest, erm I would say there is definitely a couple of people that I know that yes are a bit more oh well I am negative so I am just going to do what I want sort of thing (P102, Male, Yr5, Student)  I guess we would just be more careful if someone did test positive because like you know it is the whole you can meet one other household outside or inside, but like there is nothing stopping like that one person meeting the person who may have had it and then them meeting another household and then it is slowly doing that like little domino effect and then eventually getting back to us. So, if there was someone who had it we would probably… we might just even like isolate ourselves just to like to stay on the safe side to prevent anything from spreading further. (P91, Female, Yr1, Student)  Erm, it makes you kind of think more about who you’ve been in contact with, give people… tested positive then you’re suddenly a bit more aware of who you have met up with that week and stuff. Making sure that you are within the guidelines and not seeing more then the number of people you can be meeting with or households and things. Just in case (P104, Female, Yr1, Student)  Yes, and if there was any social events on and if there was any lectures and stuff, I would just say well you can’t come in until you have had the test. I don’t know that sounds really and it is difficult because you can’t make someone have it but at the same time, why should that person put everyone else at an increased risk? (P102, Male, Yr5, Student) |
