# Supplementary material for: Perceptions and Experiences of the University of Nottingham Pilot SARS-CoV-2 Asymptomatic Testing Service: A Mixed-Methods Study
Source: Int J Environ Res Public Health. 2020 Dec 29;18(1):188. doi: 10.3390/ijerph18010188 (PMC7796111; doi:10.3390/ijerph18010188)
Supplement: Supplementary file 1 [file ijerph-18-00188-s001.zip › Supplementary files/Supplementary File 3 Interview and Focus Group Question Guide.docx]

**Supplementary File 5.** Interview and Focus Group Questioning Guide

**Student Interviews and Focus Groups**

Introductory questions

- What are your general views on COVID and COVID testing?
- What are your views on this approach to COVID testing within the university?

Programme evaluation

- What was your main reason for taking part, or not, in this testing programme?
- What are your views on the way in which information was communicated to you at the start of, and during this testing programme?
- How did you feel about the testing process itself?
- Was there anything that impacted on your decision to do the test or not?
- Did you have any particular concerns about this testing programme?
- Do you perceive any particular benefits of being involved in this testing programme?
- How do you feel about saliva testing compared with swab tests?
- What approach do you think would be appropriate going forwards?

**Staff Focus Group**

Introductory questions

- What is your job role?
- Have you had contact with the year 1 (April) or year 5 students during the pilot programme? What was the nature of this contact?
- Have you taken part in the university testing programme yourself?
- Have you had to self-isolate during the pilot testing programme?
- What are your general views about COVID-19? Do you have any particular personal experiences with COVID, concerns or worries?

Views towards university working life during COVID-19

- How do you feel about working at a university at the moment? And students attending?

Views on university testing

- What are your general views about asymptomatic testing within a university setting? What are the potential benefits or drawbacks?

Programme Evaluation

- What was your main reason for taking part, or not?
- What went well or less well?
- How did you feel about the testing processes?
- How did you feel about the programme communications?
- What were the impacts of the programme, if any?
